# Funicular pain: a case report of intermittent claudication induced by cervical cord compression

**DOI:** 10.1186/s12891-020-03299-x

**Published:** 2020-05-14

**Authors:** Takuhei Kozaki, Akihito Minamide, Hiroshi Iwasaki, Yasutsugu Yuakawa, Muneharu Ando, Hiroshi Yamada

**Affiliations:** 1 Department of Orthopaedic Surgery, Wakayama Medical University, 811-1 Kimiidera, Wakayama City, Wakayama, 641-8509 Japan; 2 Department of Orthopaedic Surgery, Kansai Medical University, 2-3-1 Shinmachi, Hirakata City, Osaka, 573-1191 Japan

**Keywords:** Funicular pain, Tract pain, Cervical spondylotic myelopathy, Post-myelogram dynamic computed tomography, Laminoplasty, Case report

## Abstract

**Background:**

Neurogenic origin intermittent claudication is typically caused by lumbar spinal canal stenosis. However, there are few reports of intermittent claudication caused by cervical spinal cord compression.

**Case presentation:**

We present the case of a 75-year-old woman who presented with intermittent claudication. She had a history of lumbar spinal fusion surgery, but there was no sign of lumbar spinal stenosis. She also reported bilateral thigh pain on cervical extension. Electromyogram (EMG), posture-induced test, myelogram, and post-myelogram dynamic computed tomography (CT) were performed. Myelography and post-myelogram dynamic CT in the cervical extension position showed narrowing of the subarachnoid space; the patient reported pain in the front of the both thigh during the procedure. We performed an electromyogram (EMG), which implied neurogenic changes below the C5 level. Based on these results, we diagnosed cervical spinal cord compression and underwent laminoplasty at C4–6 including dome-like laminectomy, which significantly relieved the thigh pain and enabled her to walk for 40 minutes.

**Conclusions:**

In this case, funicular pain presented as leg pain, but was resolved by the decompression of the cervical spinal cord. Funicular pain has various characteristics without any upper extreme symptom. This often leads to errors in diagnosis and treatment. We avoid the misdiagnosis by evaluating post-myelogram dynamic CT compared between flexion and extension. In cases of intermittent claudication, clinicians should keep in mind that cervical cord compression could be a potential cause.

## Background

Intermittent claudication can be induced by one of two mechanisms: neurogenic or vasculogenic. Neurogenic origin intermittent claudication is typically caused by lumbar spinal canal stenosis. However, there are no reports of intermittent claudication caused by cervical spinal cord compression. We report a case of cervical spinal cord compression that presented with intermittent claudication of the frontal sides of both thighs on walking, even for 5 min. Thigh pain was also induced on cervical extension position.

## Case presentation

A 75-year-old woman presented with intermittent claudication in both thighs (right>left), along with a burning sensation for 6 months. She had undergone lumbar spinal fusion surgery for spondylolisthesis at L4–6 14 years prior and the outcome was kept good. The thigh pain with burning sensation was induced by walking or standing for 5 min. There was no muscle weakness, numbness, sensory disturbance, or hyperreflexia in the upper and lower limbs. Hoffmann’s reflex, Babinski’s sign, and straight leg raising test were all negative. The 10-s grip and release test were 20 times at both sides. There was no urinary symptom. We initially suspected radicular pain caused by lumbar lesions even though Kemp’s test was negative.

**Fig. 1:**
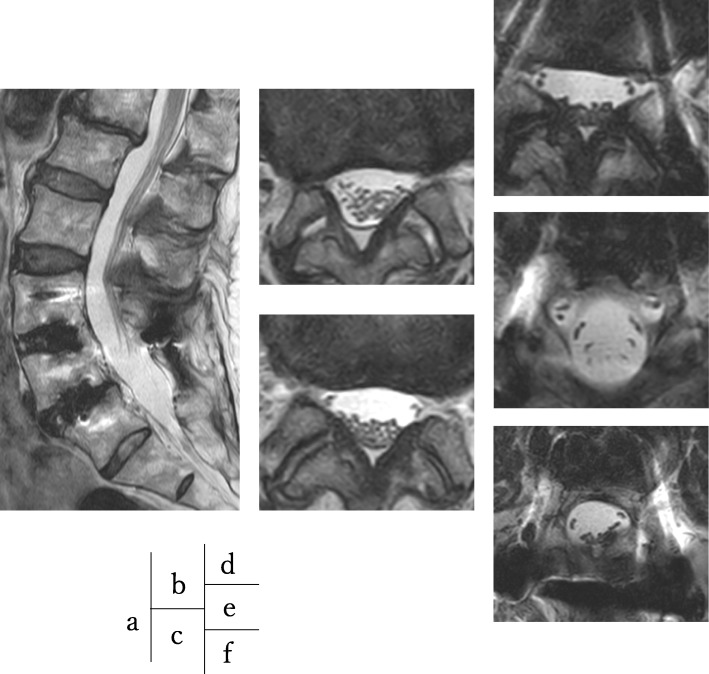
Magnetic resonance imaging at lumbar lesion. Magnetic resonance imaging showed no lumbar lesions at sagittal view (**a**) and axial view, L1/2 (**b**), L2/3 (**c**), L3/4 (**d**), L4/5 (**e**), and L5/S1 (**f**)

**Fig. 2:**
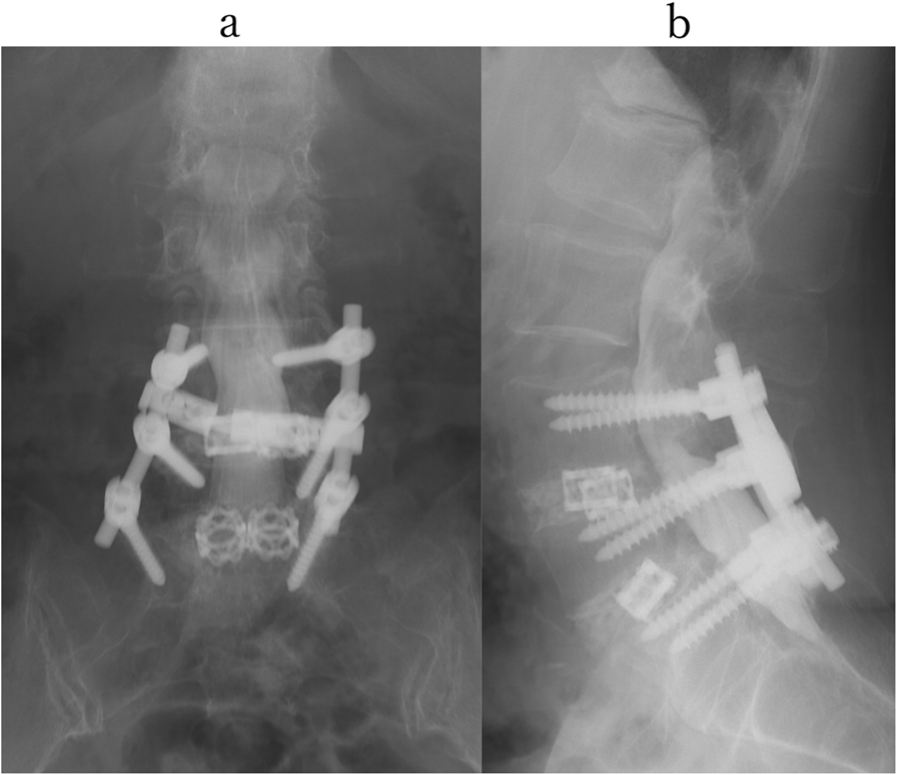
Myelography at lumber level. Myelography showed no lumbar lesions at anterior-posterior view (**a**) and lateral view (**b**)

**Fig. 3:**
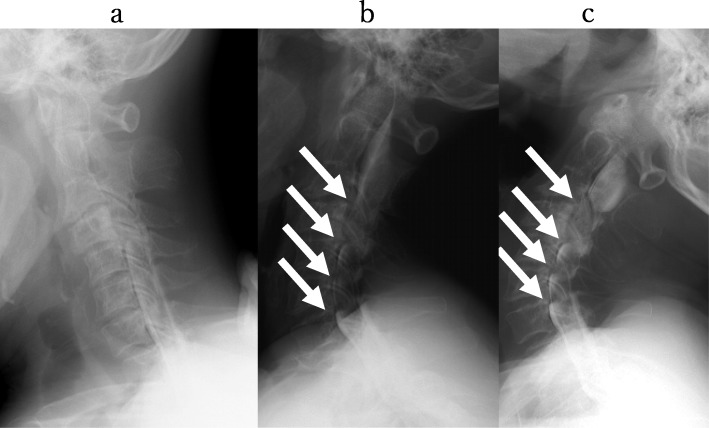
Myelography at cervical level. Myelography showed a narrower subarachnoid space at C3/4, C4/5, C5/6, and C6/7 during neutral (**b**) and extension (**c**) than flexion (**a**)

**Fig. 4:**
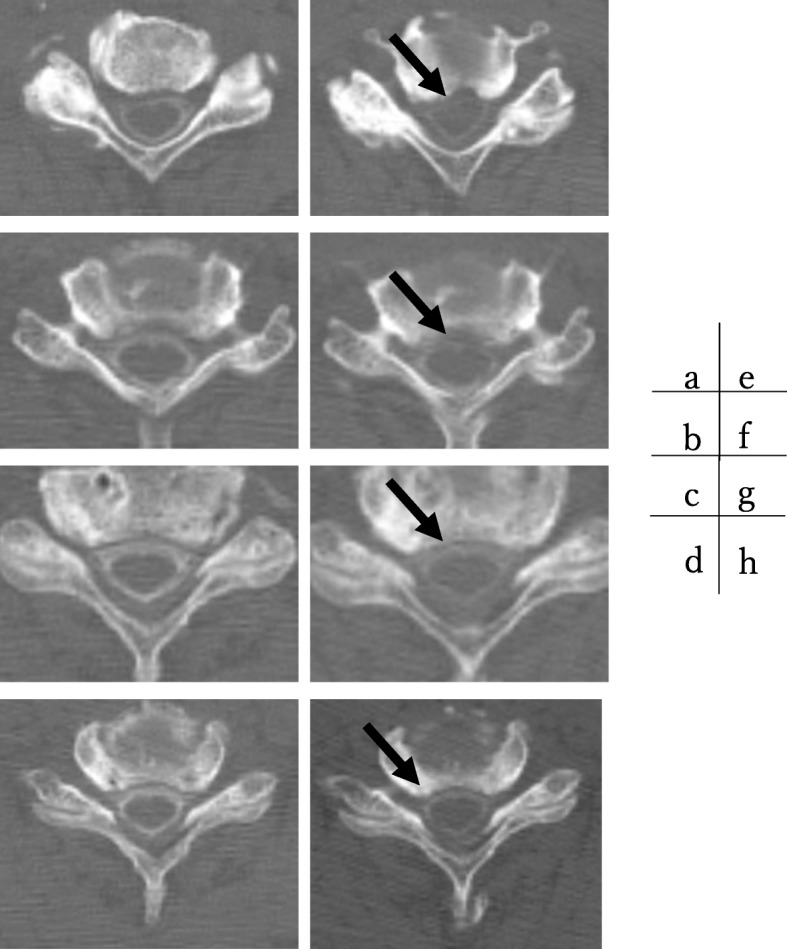
Post-myelogram dynamic computed myelography. Post-myelogram computed tomography (CT) myelography at flexion at C3/4 (**a**), C4/5 (**b**), C5/6 (**c**), and C6/7 (**d**) and extension at C3/4 (**e**), C4/5 (**f**), C5/6 (**g**) and C6/7 (**h**). CT myelography showed narrowing of the subarachnoid space at extension (e-h) compared to flexion (a-d)

**Fig. 5:**
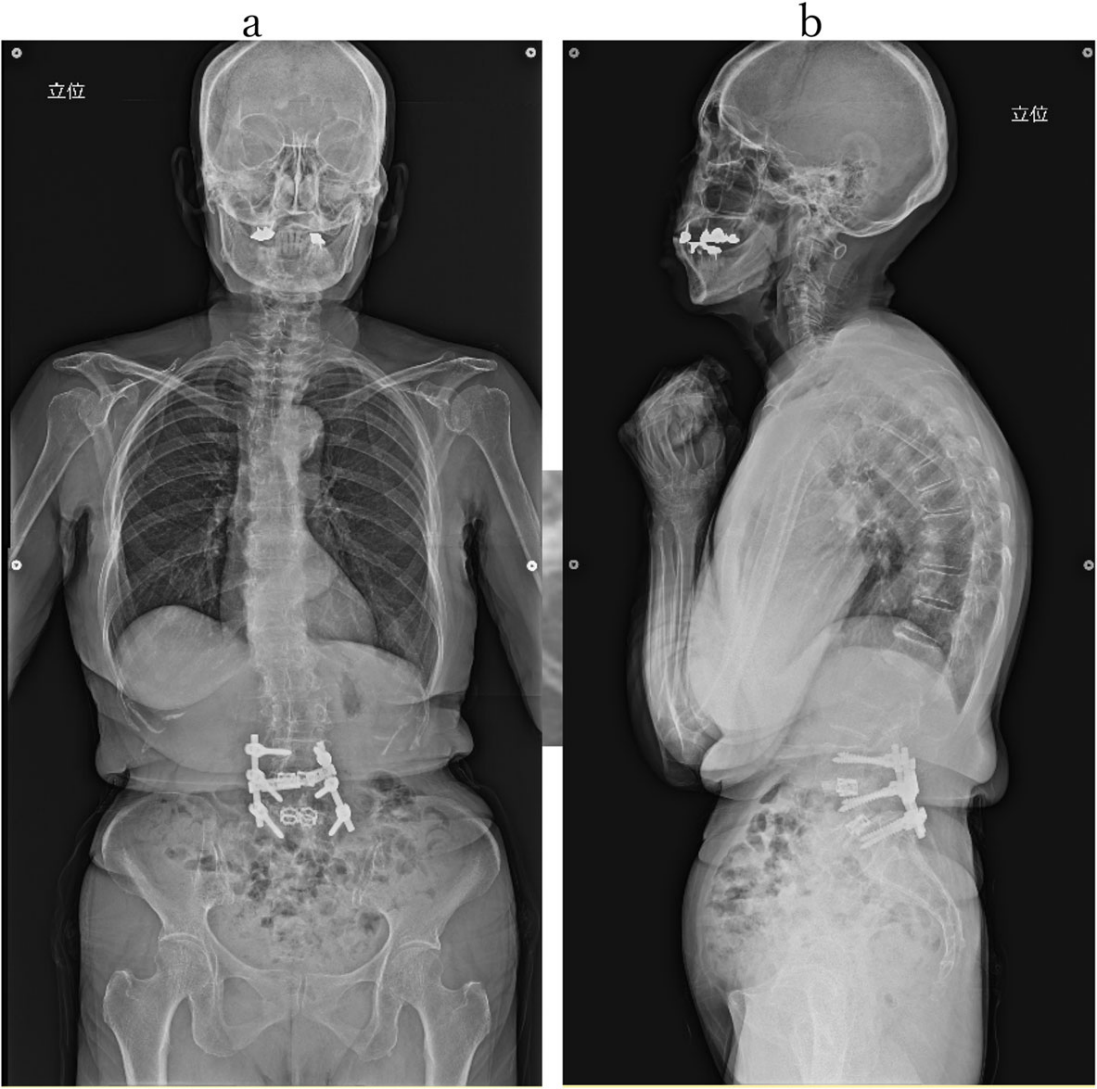
Whole spine X-rays**.** Whole spine X-rays at anterior-posterior view (**a**) and lateral view (**b**). The patient also had the slight cervical hyperlordosis following the lumbar lordosis and the thoracic increased kyphosis

However, no significant lesions were seen at the lumbar level on magnetic resonance imaging (MRI) (Fig. 1). After careful examination, thigh pain was found to be induced by cervical extension. We considered that the intermittent claudication could be caused by spinal cord compression and performed a cervical MRI, which revealed multi-level cervical spinal cord compression. Next, we performed myelography and post-myelogram dynamic computed tomography (CT) myelography of the cervical region. Myelography showed no lumbar lesions (Fig. 2); moreover, the subarachnoid space was found to be narrowed with the spine in the neutral and extension positions rather than during flexion (Fig. 3). CT myelography in the cervical extension position showed narrowing of the subarachnoid space at C3/4, 4/5, 5/6, and 6/7 levels; the patient reported pain in the front of the right thigh during the procedure (Fig. 4). As shown the myelogram on neutral and extended position, the whole spine x-rays showed the slight cervical hyperlordosis following the lumbar lordosis and the thoracic increased kyphosis (Fig. 5). We performed an electromyogram (EMG), which implied neurogenic changes below the C5 level.

**Fig. 6:**
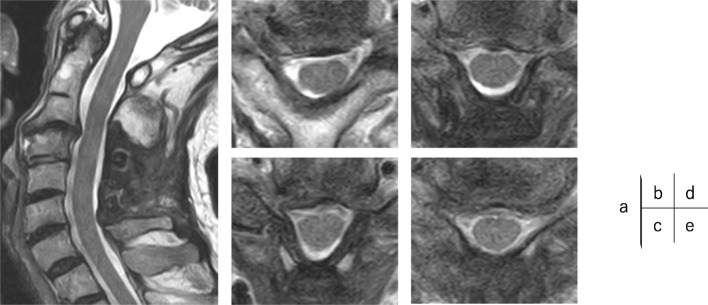
Magnetic resonance imaging at postoperation. The post-operative MRI showed enough posterior spinal shift and spinal cord decompression at  sagittal (**a**), and axial view: C3/4 (**a**), C(4/5), C(5/6), and C(6/7)

Based on these results, we diagnosed cervical spinal cord compression and performed laminoplasty at C4–6 and dome-like laminectomy at C3 and C7 because the compression of spinal cord was observed at the C3/4, C4/5, C5/6, and C6/7 levels. Postoperatively, the patient’s leg pain was completely relieved and discharged 2 weeks after the surgery. She could walk for 40 minutes continuously without thigh pain. Six months after the procedure, a repeat MRI showed adequate subarachnoid space around the spinal cord due to posterior spinal cord shift (Fig. 6). Adverse events were not recognized.

## Discussion and conclusion

We present a rare case of intermittent claudication caused by cervical spinal cord compression and resolved with laminoplasty. In this case, several false localizing signs were present; these are reported as neurological signs that do not conform to the expected anatomical level of the lesion, for example, leg pain caused by cervical cord compression [1]. Intracranial tumors are known to often cause false localizing signs [2]. It is also reported that spinal cord compression causes pain in the lower back or lower limbs below the level of the lesions; this pain often manifests as tract or funicular pain [3–8]. To the best of our knowledge, almost all cases reported till now have described leg pain without other neurological signs, which strongly suggest spinal cord compression; all cases were resolved by decompression of the spinal cord. Funicular pain was seen to present in various forms: it was continuously present [4, 7], diffuse, or the patients reported non-dermatomal and abnormal sensations [4]. None of these symptoms were correlated with the level of the lesions.

This is a very rare case; diagnosing spinal cord compression was difficult because the chief complaint was intermittent claudication, which is a major symptom of lumbar lesions and the exact pathophysiology of funicular pain had not been clarified, but irritation of the spinothalamic tract has been proposed as the cause [6]. In this case, the spinal cord on the ventral lateral side was more compressed in post-myelogram dynamic CT on the extension position than flexion. It is not cleared, but the results of myelography indicate the possibility of the irritation of the spinothalamic tract as the pathology of thigh pain on the extension and intermittent claudication.

These was a possible risk of incorrect diagnosis and unnecessary surgery because the only symptom was pain without any other neurological signs. To prevent misdiagnosis and correctly identify the cause of the symptoms, some methods are reported, such as cervical epidural blocks [7] and electrophysiological procedure [9]. Cervical interlaminar epidural blocks are reported to be effective in enabling correct identification of the responsible level by relieving the pain [7]. Electrophysiological procedures such as spinal cord-evoked potentials [9], motor-evoked potentials, and somatosensory-evoked potentials [10] are reported to be useful for diagnosing cord compression that presents with few symptoms in the upper extremities.

In this case, we conducted the posture-induced test, myelography, post-myelogram dynamic CT myelography, and electromyography to determine the cause of the symptoms. Dynamic CT myelography can provide valuable information for determining treatment strategies and objectives. The spinal cord cross-sectional area becomes narrower during extension in patients with ossification of posterior longitudinal ligament [11]. Our patient presented with thigh pain at cervical extension and the spinal cord cross-sectional area became narrower in the extension-CT myelography, with thigh pain. Whole spine X-rays on standing showed that our patient preserved lumbar lordosis and compensatory increased thoracic kyphosis and cervical lordosis, which might indicate that just standing position influence on the spinal cord as extended and neutral position on myelogram and post myelogram extended-CT. Electromyography also showed neurogenic change below the C5 level. These procedures are effective to diagnose cervical cord compression depending on the cervical position, especially if the patient does not present any typical symptoms in the upper extremities.

Here, we report the few cases of funicular pain that presented as intermittent claudication of the thighs induced by walking for 5 min. Funicular pain poses diagnostic problems, especially if no other neurological symptoms are present in the upper extremities. Clinicians should suspect possible cord compression at the upper level in patients with funicular pain irrespective of upper extremity symptoms. To prevent misdiagnosis and unnecessary surgery, several investigations can be used, including myelography and post-myelography dynamic CT myelogram. This case report also showed that dynamic CT myelography can enable the clinician to successfully identify the cause of the funicular pain depending on the posture.

## Data Availability

Not applicable.

## Abbreviations

EMG: Electromyogram
CT: Computed Tomography
MRI: Magnetic resonance imaging
